# Consequences of Hyperoxia and the Toxicity of Oxygen in the Lung

**DOI:** 10.1155/2011/260482

**Published:** 2011-06-05

**Authors:** William J. Mach, Amanda R. Thimmesch, J. Thomas Pierce, Janet D. Pierce

**Affiliations:** ^1^School of Nursing, University of Kansas, 3901 Rainbow Boulevard, Kansas City, KS 66160, USA; ^2^U.S. Department of Veterans Affairs (122), Rehabilitation Research and Development Service, 810 Vermont Avenue, NW, Washington, DC 20420, USA

## Abstract

Oxygen (O_2_) is life essential but as a drug has a maximum positive biological benefit and accompanying toxicity effects. Oxygen is therapeutic for treatment of hypoxemia and hypoxia associated with many pathological processes. Pathophysiological processes are associated with increased levels of hyperoxia-induced reactive O_2_ species (ROS) which may readily react with surrounding biological tissues, damaging lipids, proteins, and nucleic acids. Protective antioxidant defenses can become overwhelmed with ROS leading to oxidative stress. Activated alveolar capillary endothelium is characterized by increased adhesiveness causing accumulation of cell populations such as neutrophils, which are a source of ROS. Increased levels of ROS cause hyperpermeability, coagulopathy, and collagen deposition as well as other irreversible changes occurring within the alveolar space. In hyperoxia, multiple signaling pathways determine the pulmonary cellular response: apoptosis, necrosis, or repair. Understanding the effects of O_2_ administration is important to prevent inadvertent alveolar damage caused by hyperoxia in patients requiring supplemental oxygenation.

## 1. Introduction

When administering supplemental oxygen (O_2_) to treat hypoxemia associated with acute and chronic conditions, O_2_ toxicity by overexposure may be present. Annually, the need for supplemental O_2_ is projected to be around 800,000 individuals at a cost of 1.8 billion dollars [1]. Suboptimal use of O_2_ is reflected in prescription and treatment errors that exceed those related to antibiotics [2–4]. 

The alveolar epithelial and alveolar capillary endothelial cells are vulnerable targets for O_2_-free-radical-induced injury caused by hyperoxia. In acute lung injury (ALI) caused by hyperoxia, hyperpermeability of the pulmonary microvasculature causes flooding of the alveolus with plasma extravasations leading to pulmonary edema and abnormalities in the coagulation and fibrinolysis pathways promoting fibrin deposition [5, 6]. Type II alveolar epithelial cells are injured by O_2_ free radicals leading to impairment of surfactant production [7]. Thus, the maximum positive biological benefit for this life essential but toxic molecule exists along a dose-response, deficiency–toxicity continuum.

## 2. Pathophysiology of Oxygen Toxicity

Hyperoxia is a state of excess supply of O_2_ in tissues and organs. Oxygen toxicity occurs when the partial pressure of alveolar O_2_ (P_A_O_2_) exceeds that which is breathed under normal conditions. With continuous exposure to supraphysiologic concentrations of O_2_, a state of hyperoxia develops. Under hyperoxic pathological conditions, a large influx of reactive O_2_ species (ROS) are produced. In intracellular and extracellular biological systems, the mass effect of ROS elevation, caused by O_2_ overexposure, disrupts the balance between oxidants and antioxidants, and this disruption of homeostasis can result in damage to cells and tissues [8–11]. 

Exposure time, atmospheric pressure, and fraction of inspired O_2_ (FIO_2_) determine the cumulative O_2_ dose leading to toxicity. Oxygen is toxic to the lungs when high FIO_2_ (>0.60) is administered over extended exposure time (≥24 hours) at normal barometric pressure (1 atmospheres absolute (ATA)). This type of exposure is referred to as low pressure O_2_ poisoning, pulmonary toxicity, or the Lorraine Smith effect. Oxygen exposure after approximately 12 hours leads to lung passageway congestion, pulmonary edema, and atelectasis caused by damage to the linings of the bronchi and alveoli. The formation of fluid in the lungs causes a feeling of shortness of breath combined with a burning of the throat and chest, and breathing becomes very painful [12]. The reason for this effect in the lungs but not in other tissues is that the air spaces of the lungs are directly exposed to the high O_2_ pressure. Oxygen is delivered to the other body tissues at almost normal partial pressure of O_2_ (PO_2_) because of the hemoglobin-O_2_ buffer system [13–15]. Toxicity also occurs when the ATA is high (1.6–4) and the high FIO_2_ exposure time is short. This type of exposure is referred to as high pressure O_2_ poisoning or the Paul Bert effect and is toxic to the central nervous system (CNS). Central nervous system toxicity results in seizures followed by coma in most people within 30 to 60 minutes. Seizures often occur without warning and are likely to be lethal. Other symptoms include nausea, muscle twitching, dizziness, disturbances of vision, irritability, and disorientation [13, 16–20]. Oceanic divers are more likely to experience CNS toxicity [17].

Pulmonary capillary endothelial and alveolar epithelial cells are targets for ROS resulting in injury-induced lung edema, alveolar flooding, hemorrhage, and collagen, elastin, and hyaline membrane deposits [11, 21, 22]. Above a critical P_A_O_2_, the hemoglobin-O_2_ buffering mechanism fails and the tissue PO_2_ can rise to hundreds or thousands of mm Hg. At high levels of O_2_, protective endogenous antioxidant enzyme systems become consumed by ROS leading to cell death [16, 23]. 

Oxygen toxicity caused by ROS progresses in overlapping phases based on degree of severity and reversibility of injury. The phases are initiation, inflammation, proliferation, and fibrosis. Initially, there are increased ROS and depleted antioxidant levels, and the lung fails to clear itself of mucous. The inflammation phase or exudative phase is characterized by the destruction of the pulmonary lining and migration of leukocyte derived inflammatory mediators to the sites of injury. The proliferative phase is subacute and there are cellular hypertrophy, increased secretions from surfactant secreting alveolar type II cells, and increased monocytes. The final terminal phase is the fibrotic phase in which the changes to the lung are irreversible and permanent. There is collagen deposition and thickening of the pulmonary interstitial space and the lung becomes fibrotic [24–27].

Clinically, progressive hypoxemia, or high O_2_ tension in the blood, requires increased FIO_2_ and assisted ventilation, which further aggravate the pathophysiological changes associated with O_2_ toxicity. Chest X-rays may show an alveolar interstitial pattern in an irregular distribution with evidence of a moderate loss of volume from atelectasis, however there is no clinical way of diagnosing O_2_ toxicity. Lung biopsy specimens may show changes consistent with O_2_ toxicity but the primary value of the biopsy is to exclude other causes of lung injury. Air pressure changes within the enclosed lung cavity and ventilator-induced injury may accompany and be indistinguishable from O_2_ toxicity. Oxygen toxicity can be minimized by keeping the P_A_O_2_ less than 80 mm Hg or the FIO_2_ below 0.40 to 0.50 [12]. 

The pulmonary cellular response to hyperoxic exposure and increased ROS is well described. Anatomically, the pulmonary epithelial surface is vulnerable to a destructive inflammatory response. This inflammation damages the alveolar capillary barrier leading to impaired gas exchange and pulmonary edema. Reactive O_2_ species induces pulmonary cell secretion of chemoattractants, and cytokines stimulate macrophage and monocyte mobilization and accumulation into the lungs, leading to additional ROS. The ROS leukocyte interaction further exacerbates injury. Research has shown that as these highly reduced cell layers become increasingly oxidized and levels of antioxidants fall, ROS-induced activation of multiple upstream signal transduction pathways regulates the cellular response: adaptation, repair, or cell death by apoptosis, oncosis, or necrosis [28, 29]. 

Mitogen-activated protein kinase (MAPK), toll-like receptor 4 (TLR4), signal transducers and activators of transcription (STAT), and nuclear factor kappa beta (NF k*β*) are a few well-researched protein pathways that communicate the receptor signal to the deoxyribonucleic acid (DNA) of the cell thereby determining the cellular response. The MAPK pathway is a regulator of cell death genes, stress, and transformation and growth regulation. Mitogen-activated protein kinase activation precedes extracellular signal regulated kinase (ERK1/2), a promoter of cell proliferation. C-Jun-terminal protein kinase (JNK1/2) and p38 kinase both induce cell death and inflammation [30]. The TLR4, STAT, and nuclear regulatory factor 2 (Nrf2) pathways are associated with survival gene expression such as caspase-3 proteins and antioxidant response element (ARE) [31, 32]. The NF k*β* pathway is an up-stream signal for inflammation and survival genes: anti-oxidant enzymes (AOE), Bcl-2, AKT, heme oxygenase (HO-1), and heat shock proteins (HSPs). The AKT_1-4_ family of signals plays an important role in glucose metabolism, cell proliferation, apoptosis, transcription, and cell migration. The Bcl-2 proteins are antiapoptotic while HO-1 and HSPs are ubiquitous stress-response proteins [33]. These signaling pathways are regulators of the pulmonary epithelial cell response to increases in ROS and hyperoxia [18, 34]. Cytokine and chemokine overexpression in response to hyperoxic stress can be protective. Tumor necrosis factor alpha (TNF*α*), interleukin 1 beta (IL-1*β*), interleukin 6 (IL-6), chemokine receptor 2 (CXCR2), interleukin 11 (IL-11), insulin and keratinocyte growth factor expression, and the beta subunit of Na, K-ATPase have been shown to attenuate death signals [35–37].

## 3. The Formation of Free Radicals

Oxygen is a requirement for cellular respiration in the metabolism of glucose and the majority of O_2_ consumed by the mitochondria is utilized for adenosine triphosphate (ATP) generation [38, 39]. The mitochondrial electron transport chain reduces the elemental molecular O_2_ to ionic O_2_ by the relay of electrons making O_2_ usable for ATP generation, during this process, oxidizing free radicals are generated [40, 41]. Toxic levels of O_2_ lead to the formation of additional ROS, which can impose damage to lipid membranes, proteins, and nucleic acids. Reactive O_2_ species mediate physiological and pathophysiological roles within the body [42]. 

**Figure 1:**
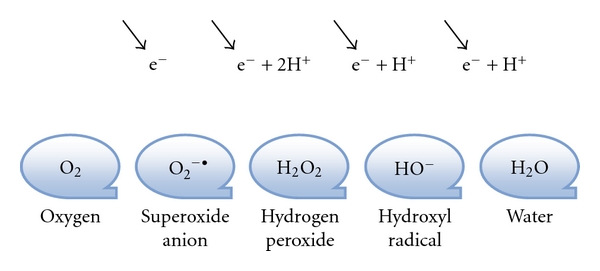
Reduction of oxygen. A single-electron transfer which converts molecular oxygen to the superoxide anion, creating an unstable molecule. The decomposition of hydrogen peroxide can be a source of the hydroxyl radical; this reaction requires both superoxide and hydrogen peroxide as precursors. These steps reduce oxygen to water by the addition of four electrons, yielding three reactive oxygen species: superoxide anion, hydrogen peroxide, and hydroxyl radical.

Free radicals are a type of unstable, reactive, short-lived chemical species that have one or more unpaired electrons and may possess a net charge or be neutral. The species is termed free because the unpaired electron in the outer orbit is free to interact with surrounding molecules [42, 43]. Cells generate free radicals, or ROS, by the reduction of molecular O_2_ to water (H_2_O) (Figure 1) [44, 45]. 

Chemically, three types of reactions lead to the formation of ROS. The one-electron reduction of molecular O_2_ to the superoxide anion (O_2_^−●^) is catalyzed by transition metals including iron (Fe) and copper (Cu) such as

(1) $${\text{Fe}}^{\text{II}}+{\text{O}}_{2}\to {\text{Fe}}^{\text{III}}+{{\text{O}}_{2}}^{-●}.$$

 The simultaneous oxidation reduction reaction of O_2_^−●^ to hydrogen peroxide (H_2_O_2_) and the addition of an electron to O_2_^−●^ produce the hydroxyl radical (HO^●^). The O_2_^−●^ in biological membranes can act in four different modes: electron transfer, nucleophilic substitution, deprotonation, and a hydrogen atom abstraction as in

(2) $${{\text{O}}_{2}}^{-●}\;+\;{{\text{O}}_{2}}^{-●}\;+\;{2\text{H}}^{+}\to {\text{H}}_{2}{\text{O}}_{2}+{\text{O}}_{2}.$$

 A  O_2_^−●^ initiated Fenton-type reaction and the decomposition of H_2_O_2_ requires O_2_^−●^ and H_2_O_2_ as precursors and Fe and Cu presence for completion. The HO^●^ is the most injury producing in biological systems, reacting with molecules in close proximity. These reactions are called Fenton-like reactions generating O_2_ and HO^●^ when Fe^ II ^ or Cu^ I ^ reacts with H_2_O_2_

(3) $${\text{Fe}}^{\text{III}}+{{\text{O}}_{2}}^{-●}\;\to {\text{Fe}}^{\text{II}}+{\text{O}}_{2}$$

(4) $${\text{Fe}}^{\text{II}}\;+\;{\text{H}}_{2}{\text{O}}_{2}\to {\text{Fe}}^{\text{III}}+{\text{HO}}^{●}+{\text{HO}}^{●}$$

(5) $${{\text{O}}_{2}}^{-●}+{\text{H}}_{2}{\text{O}}_{2}\to \;{\text{O}}_{2}+{\text{HO}}^{●}+\;{\text{HO}}^{●}.$$

The sum of reactions (3) and (4), or the Haber-Weiss reaction shown in (5) above demonstrates HO^●^ formation by the metal-catalyzed decomposition of H_2_O_2_. The interaction between O_2_^−●^ and H_2_O_2_ is the source of the majority of damage to biological systems due to the reactivity of continuously produced, highly toxic HO^●^ [18, 46, 47]. These ROS-producing reactions occur endogenously involving enzymes, neutrophils, and organelles such as the mitochondria and exogenously induced by radiation, pollutants, xenobiotics, and toxins. Cellular survival and adaptation in an oxidative atmosphere are dependent upon sufficient antioxidant defenses to counteract the effects of ROS on cells and tissues [48].

## 4. Functions and Classifications of Antioxidants

Oxidant antioxidant homeostasis is highly regulated and essential for maintaining cellular and biochemical functions [49]. A change in the balance toward an increase in the oxidant over the capacity of the antioxidant defines oxidative stress and can lead to oxidative damage. Changing the balance toward an increase in the reducing power of the antioxidant can also cause damage and is defined as reductive stress [50–52]. Reduction, antioxidant and oxidation, or pro-oxidant reactions result from a gain or a loss of electrons and a loss or a gain in O_2_ [50, 53, 54].

An antioxidant (a reductant or reducing agent) is anything that can prevent or inhibit oxidation [55–57]. Delay of oxidation can be achieved by preventing the generation or inactivating ROS [58]. Prevention, diversion, dismutation (decay), scavenging, and quenching are specialized antioxidant properties (Table 1). Antioxidant defenses may be classified as nonenzymatic and enzymatic or endogenous and dietary. Examples of nonenzymatic antioxidants are glutathione (GSH), ascorbic acid, vitamin E, beta-carotene, and uric acid. Major enzymatic antioxidants are superoxide dismutase (SOD), catalase, and GSH peroxidase which divert or dismutate ROS into harmless products. Endogenous or dietary antioxidants are based on the ability of the antioxidant to be synthesized by humans. Endogenous antioxidants are SOD, catalase, GSH peroxidase, uric acid, and bilirubin. Dietary antioxidants are ascorbic acid, vitamin E, and beta-carotene [59, 60]. Ascorbic acid, vitamin E, uric acid, bilirubin, and GSH scavenge ROS by expendable, replaceable, or recyclable substrates. Vitamin E and beta-carotene quench ROS by absorption of electrons and/or energy.

Antioxidants can be classified into four categories based on function. (1) Preventive antioxidants which suppress formation of ROS, (2) radical scavenging antioxidants which suppress chain initiation and/or break chain propagation reactions, (3) the repair and de novo antioxidants such as proteolytic enzymes and the repair enzymes of DNA, and (4) antioxidants which allow for adaptation that occurs when the signal for the production and reactions of ROS induces oxidant formation and transport [10, 61].

Superoxide dismutase converts O_2_^●−^ to H_2_O_2_ and has three isoforms widely distributed in mammalian organisms. (1) Cytoplasmic SOD (SOD1 or Cu zinc (CuZn) SOD) is located in the cytoplasm, nucleus, and peroxisomes, (2) mitochondrial SOD (SOD2 or MnSOD) is located in the mitochondrial matrix near the electron transport chain, and (3) extracellular SOD (SOD3 or EcSOD) is found in the extracellular fluids and extracellular matrix of all human tissues especially the heart, placenta, pancreas, and lung [62–64]. The protective effects of EcSOD in the lungs are extremely important and well-established [65–68]. 

Catalase, one of the most potent catalysts found mostly in the peroxisome, functions to decompose H_2_O_2_ to H_2_O. Catalase defense from oxidant injury to lung epithelial cells exists in the cytosol or the mitochondria. 

Glutathione reductase is an important antioxidant enzyme for maintaining the intracellular reducing environment. This enzyme catalyzes the reduction of glutathione disulfide (GSSG) to GSH [69]. Glutathione disulfide is produced through the oxidation of GSH by ROS that arise during conditions of oxidative stress. Due to the high concentrations of GSH, GSH/GSSG is considered to be the principal redox buffer of the cell and the ratio of GSH/GSSG is viewed as a major indicator of the cellular redox status. The ratio of GSH/GSSG decreases under an oxidative stress condition [70, 71]. Tissue damage may develop when an oxidant/antioxidant imbalance occurs as a consequence of hyperoxia [72, 73]. The damaging effects of hyperoxia can lead to O_2_ toxicity, cell death, and can be a triggering factor in ALI [22].

## 5. Clinical Presentation of Hyperoxic Acute Lung Injury

Acute lung injury and acute respiratory distress syndrome (ARDS) are secondarily occurring, inflammatory syndromes caused by triggers or risk factors described as direct or indirect, pulmonary or extrapulmonary. The pathological changes associated with HALI mimic the ALI triggered by other conditions such as hemorrhagic shock, reperfusion injury, pneumonia, sepsis, or paraquat inhalation [23, 33, 74, 75]. The risk of developing ALI or ARDS after inhalation injury is dependent on the toxicity and concentration of the inhaled substance [17]. For example, the cells and structure of the alveolar capillary membrane are highly susceptible to damage by toxic levels of O_2 _ [76]. Both ALI and ARDS are the same clinical disorder, differing only in severity of hypoxemia. The ratio between arterial pressure of O_2_ (PaO_2_) and the FIO_2_ concentration delivered by ventilator support distinguishes the two syndromes. For ALI, the PaO_2_/FIO_2_ is ≤300 mm Hg and for ARDS, the PaO_2_/FIO_2_ is ≤200 mm Hg [74, 75, 77]. 

The injury to the alveolus is thought to develop when pulmonary or systemic inflammation leads to systemic release of cytokines and other proinflammatory molecules. Mast cells, which express mediators that exert effects on lung vasculature, are also increased after hyperoxic exposure [78]. Cytokine release activates alveolar macrophages and recruits neutrophils to the lungs. Subsequent activation of leukotrienes, oxidants, platelet activating factor, and protease occurs. These substances damage capillary endothelium and alveolar epithelium, disrupting the barriers between the capillaries and air spaces. Edema fluid, proteins, and cellular debris flood the air spaces and interstitium, causing disruption of surfactant, airspace collapse, ventilation-perfusion mismatch, shunting, and stiffening of the lungs with decreased compliance and pulmonary hypertension. There is no pattern to the injury; however, dependant lung areas are most frequently affected [74, 79]. 

Tissue examination reveals that surfactant disruption, epithelial injury, and sepsis initiate the increased expression of cytokines that sequester and activate inflammatory cells. Increased release of ROS alters normal endothelial function. Microarray analysis has revealed increased expression of genes related to oxidative stress, antiproteolytic function, and extracellular matrix repair as well as decreased surfactant proteins in ozone-induced ALI [80]. Diffuse alveolar damage results with intra-alveolar neutrophils indicating the presence of an inflammatory response in the alveoli. Red blood cells, cellular fragments, and eroded epithelial basement membranes are present with formation of hyaline membranes, indicating that serum proteins have entered and precipitated in the air spaces due to disruption of the alveolar capillary barrier. Formation of microthrombi indicates the presence of endothelial injury and activation of the coagulation cascade [81]. 

Acute lung injury syndrome presents within 24 to 48 hours after the direct or indirect trigger. Initially, the patient may experience dyspnea, cough, chest pain, tachypnea, tachycardia, accessory muscle use, cyanosis, mottled skin, and abnormal breath sounds (crackles, rhonchi, and wheezing). Blood gas analysis reveals progressive worsening of hypoxemia, leading to respiratory failure. Bilateral infiltrates are seen on a chest X-ray and are consistent with pulmonary edema but without the cardiac component of elevated left atrial pressure. Treatment includes mechanical ventilation, supportive care, and treatment of the underlying causes [16]. The mortality of ALI has improved over the past decade; however, it still ranges from 30% to 75% [75, 77, 82, 83] and occurs in about 86 of 100,000 individuals per year [84].

## 6. Conclusion

Oxygen, often used to treat hypoxemia in the clinical setting, is itself a triggering factor in HALI given that the exposure is sufficiently concentrated and of adequate duration. The lung is a vulnerable target for oxidant-induced injury, initiating a cascade of protein signals that determine the cellular response. The alveolar epithelial and alveolar capillary endothelial surfaces are injured. Hyperpermeability, microthrombi (resulting from altered coagulation and fibrinolysis), collagen deposition, and fibrosis alter alveolar structure and function. Understanding precise mechanisms of injury and pulmonary cellular responses to hyperoxia is essential evidence for expert practice.

## Figures and Tables

**Table 1:** Locations and properties of antioxidants.

Enzymatic antioxidants located in mitochondria and cytosol	
Glutathione peroxidase (GSH)	Removal of H_2_O_2_, hydroperoxides
Superoxide dismutase (SOD)	Catalytic removal of O_2_
Catalase (CAT)	Catalytic reduction of H_2_O_2_ to H_2_O

Nonenzymatic antioxidants located in cell membrane, exogenous dietary source	
Vitamin E (*α* tocopherol)	Chain-breaking antioxidant
*β*-carotene	Scavenger of ROS, singlet O_2_ quencher
Co-enzyme Q	Regenerates vitamin E

Compounds that reduce the availability of transition metals, Fenton reactions	
Transferrin	Sequesters iron and copper ions
Lactoferrin	Sequesters iron at lower pH
Albumin	Sequesters heme and copper
Ceruloplasmin (ferroxidase)	Scavenges superoxide radical, binds copper ions

Scavengers, products of metabolism, exogenous dietary source	
Bilirubin	Scavenges peroxyl radical
Uric acid	Scavenges hydroxyl radical
Vitamin C (ascorbic acid)	Scavenges hydroxyl radical, recycles vitamin E

Thiol group donors	
Reduced glutathione (GSSH)	Binds free radicals, SH group oxidized to disulfide group (GSSG)
*α*-lipoic acid	Recycles vitamin C, glutathione substitute
